# Integrative Effects of Feeding *Aspergillus awamori* and *fructooligosaccharide* on Growth Performance and Digestibility in Broilers: Promotion Muscle Protein Metabolism

**DOI:** 10.1155/2014/946859

**Published:** 2014-05-04

**Authors:** Ahmed A. Saleh, Khairy Amber, Mohammed A. El-Magd, Mostafa S. Atta, Ahmed A. Mohammed, Mohamed M. Ragab, Hanaa Abd El-Kader

**Affiliations:** ^1^Department of Poultry Production, Faculty of Agriculture, Kafrelsheikh University, Kafrelsheikh 333516, Egypt; ^2^Anatomy Department, Faculty of Veterinary Medicine, Kafrelsheikh University, Kafrelsheikh, Egypt; ^3^Physiology Department, Faculty of Veterinary Medicine, Kafrelsheikh University, Kafrelsheikh, Egypt; ^4^Animal Health Research Institute, Giza, Egypt

## Abstract

This study was conducted to show the effect of *Aspergillus awamori* (AA), *fructooligosaccharide* (FOS), and combined *Aspergillus awamori* and *fructooligosaccharide* (AA + FOS) on growth, digestibility, blood parameters, and expression of some growth-related genes. A total of 60 broiler chicks at the age of 15 d were divided into a control group (*n* = 15) and 3 treatment groups. The control group was fed a basal diet, and the treatment groups were fed basal diets supplemented with 0.05% AA, 0.05% FOS, and combined of 0.05% AA and 0.05% FOS. Results from measurement of growth performance and digestibility revealed a significant increase in the body weight gain with improved feed conversion rate in the experimental groups. Interestingly, dry matter digestibility (DMD) and crude protein utilization (CPU) were improved. In addition, plasma total cholesterol and low density lipoprotein-cholesterol (LDL-C) were decreased, while plasma high density lipoprotein-cholesterol (HDL-C) was increased by feeding AA, FOS, and AA + FOS. Expressions of growth hormone secretagogue receptor (*GHSR*), insulin-like growth factor 1 (*IGF-1*), and insulin-like growth factor 1 receptor (*IGF1R*) were increased in experimental groups. In conclusion, the supplementation of either *Aspergillus awamori* or *fructooligosaccharide* or both improves digestibility and growth performance probably by promoting skeletal muscle protein metabolism.

## 1. Introduction

Probiotics are live cultures of organisms supplemented in animal diets that can beneficially affect the host animal by improving the microbial balance in the gut [1]. Recently,* Aspergillus* species (*A. oryzae, A. niger,* and* A. awamori*) are used as probiotics [2–4].* Aspergillus* species are extensively used for industrial enzyme production (mainly a-amylases and glucoamylases) due to their superior ability to secrete enzymes [5]. Saleh et al. reported that* Aspergillus awamori* is a fungus used for food processing in Japan [2]. The products processed by* A. awamori* are given generally recognized as safe (GRAS) status from Food and Drug Administration (FDA) [6]. The physiological effects related to* Aspergillus* probiotic include the reduction of gut pH, production of some digestive enzymes and vitamins, production of antibacterial substances (e.g., organic acids, bacteriocins, hydrogen peroxide, diacetyl, acetaldehyde, lactoperoxidase system, lactones, and other unidentified substances), reduction of cholesterol level in the blood, stimulation of immune system, suppression of bacterial infections, removal of carcinogens, improvement of calcium absorption, and reduction of faecal enzyme activity as well as reconstruction of normal intestinal microflora disorders caused by diarrhoeas, antibiotic therapy, and radiotherapy [7].

Prebiotics have been described as nondigestible food substances that selectively stimulate the growth of favorable species of bacteria in the gut, thereby benefitting the host [8]. These substances are primarily derived from nondigestible oligosaccharides [9]. Oligofructose, fructooligosaccharide, and inulin are examples that have been used as prebiotics [10].* Fructooligosaccharides* can be used as alternative of antibiotics to enhance the growth and production efficiency of broilers [11] and they are classified as nondigestible oligosaccharides because the **β**-linkages between fructose monomers cannot be hydrolyzed by enzymes of endogenous origin [12]. The physiological effects related to* Fructooligosaccharides* have been shown to enhance the growth of* Bifidobacterium* and* Lactobacillus* but inhibit* Escherichia coli* and* Salmonella* in the large intestine [13, 14].

The combination of prebiotic and probiotic is referred as synbiotic [15]. It has been proposed that synbiotics are strategically beneficial for the broilers by improving the survival rate and colonization of the introduced probiotic microorganisms in the gastrointestinal tract. At the same time, the presence of prebiotics provides a readily available substrate for probiotic growth and may promote the metabolism of the beneficial bacteria [16].

The growth and development of chickens are primarily regulated by genes of the somatotropic axis. Ghrelin receptor, or growth hormone secretagogue receptor (*GHSR*), insulin-like growth factor 1 (*IGF1*), and insulin-like growth factor 1 receptor (*IGF1R*) are important genes of this axis which have a significant role in muscles development and growth. The* IGF1* gene stimulates glucose uptake, amino acid uptake, and protein synthesis and inhibits protein degradation by satellite cell-derived myotubes [17]. Also, IGF1 protein and mRNA expression are induced under conditions of increased muscle growth and regeneration [18]. Indeed, Mitchell et al. reported that overexpression of the* IGF1* gene in the muscle tissue leads to enhanced muscle growth in chicks [19]. On the other hand, Sun et al. found that GHSR plays an essential role in energy expenditure, food intake, and food conversion rate [20]. Mice lack GHSR, consume less food, have low food conversion rate, preferentially utilize fat as an energy substrate, and have less fat content and less body weight than the control mice [21]. We hypothesized that the supplementation of either AA or FOS or both improves growth performance by the following two mechanisms: enhancing muscle growth and this action could be partly mediated by IGF1 and its receptor IGF1R, and increasing digestibility and subsequently the food conversion rate and this action may be regulated by GHSR.

As very few investigations about the synbiotics have been undertaken on broilers to date, the trial reported here was conducted to determine the effects of dietary* Aspergillus awamori* as probiotic and* fructooligosaccharide* as prebiotic and the combination of them as synbiotic on growth performance, some blood parameters, digestibility, and expression of some growth related-genes of broiler chicks.

## 2. Materials and Methods

### 2.1. Birds and Diets

This experiment was conducted in accordance with the guidelines of the Department of Poultry Production, Faculty of Agriculture, Kafrelsheikh University, Egypt.

Sixty, one-day-old unsexed Lohmann broilers were housed in an electrically heated battery brooder and provided with water and commercial starter diet (corn and soybean meal based diet containing 23% crude protein (CP) and metabolizable energy (ME) 13.39 MJ/kg) until 12 days of age. The chicks were housed in individual cages and fed the basal diet from 12 to 15 days of age. The composition of the basal diet (CP 21.65%, ME 13.17 MJ/kg) is shown in Table 1. Chicks were divided into four groups: a control group and three treatment groups (*n* = 15). The control group was fed a basal diet, and the experimental treatment groups were fed the basal diets supplemented with 0.05%* Aspergillus awamori* (AA) as probiotic, 0.05%* fructooligosaccharide* (FOS) as prebiotic, and combined 0.05%* A. awamori* and 0.05%* fructooligosaccharide* (AA + FOS) as synbiotic. The birds were given the experimental diets from 15 to 37 days of age. The experiment was conducted in a normal room with 14 h light: 10 h dark cycle. Room temperature was maintained at 23–25°C with relative humidity from 50 to 70% throughout the experiment. All experiments were performed in accordance with institutional guidelines concerning animal use.

### 2.2. Sampling

Body weight was recorded every 3 days and feed intake was recorded daily during the experimental period. At the end of the experimental period, the birds were weighted and slaughtered then dissected to measure the weights of breast muscle, liver, and abdominal fat. Blood samples were collected in heparinized test tubes and quickly centrifuged (3,000 rpm for 20 min) to separate the plasma. Plasma and meat samples were stored at −20°C and −8°C, respectively, until further analysis.

### 2.3. Nitrogen Retention, Crude Fiber, and Ether Extract

Utilization coefficients of nutrients were calculated for dry matter (DM), crude protein (CP), crude fiber (CF), and ether extracts (EE) by analysing the diets and collecting faces compassion for the last three days of the experiment; excreta were collected and weighted from each bird. Then the samples were dried by the drying oven and grinded. The crude protein, crude fiber, and ether extracts were analysed. The calculations were as follows: nitrogen retention (%) = (total nitrogen intake − total nitrogen excreted)/total nitrogen intake × 100; the same methods were used for all coefficients of nutrients.

### 2.4. Biochemical Analysis

Total cholesterol level, triglyceride, HDL-C, and LDL-C were measured calorimetrically using commercial kits (Diamond Diagnostics, Egypt) according to the procedure outlined by the manufacturer.

### 2.5. RT-PCR

Each muscle sample was homogenized and a total RNA was extracted using total RNA purification kit following the manufacturer protocol (Fermentas, K0731, Thermo Fisher Scientific, USA). The extracted total RNA (5 *μ*g per sample) was reverse transcribed into cDNA using Revert Aid H minus Reverse Transcriptase and as described by manufacturer (Fermentas, EP0451, Thermo Fisher Scientific, USA). The* IGF1* forward primer 5′AGCTGTTCGAATGATGGTGTTT3′ and reverse primer 5′GCCCCAGCATTCTCTTTCCTT3′,* IGF1R* forward primer 5′TCCAACACAACACTGAAGAATC3′ and reverse primer 5′ACCATATTCCAGCTATTGGAGC3′,* GHSR* forward primer 5′GTCGCCTGCGTCCTCCTCTT3′ and reverse primer 5′ACGGGCAGGAAAAAGAAGATG3′*, GAPDH* forward primer 5′GTGAAAGTCGGAGTCAACGG3′ and reverse primer 5′ACAGTGCCCTTGAAGTGTCC3′ were used to amplify partial clones of* IGF1*,* IGF1R*,* GHSR*, and the house-keeping gene* GAPDH*, respectively. The PCR was carried out in a reaction volume of 25 *μ*L, containing 1.0 *μ*L cDNA template (approximately 50 ng), 0.5 *μ*L (0.20 mM) dNTP, 2.5 *μ*L buffer, 1.5 *μ*L (2.5 mM) MgCl_2_, 1.0 *μ*L 10 *μ*moL/L forward primer, 1.0 *μ*L 10 *μ*moL/L reverse primer, 0.5 *μ*L Taq DNA polymerase (5 U/*μ*L, Fermentas, K1071, Thermo Fisher Scientific, USA), and 17.0 *μ*L nuclease free water. Thermal cycling parameters were as follows: initial denaturation at 94°C for 5 min, 35 cycles of amplification (94°C for 30 s for DNA denaturation, 55–62°C for 40 s annealing temperatures, extension at 72°C for 1 min), and final extension at 72°C for 5 min.

Following amplification, PCR products were electrophoresed and the level of expression of different bands was analyzed by an ImageJ gel analysis program [22]. This relies on comparing the density of each target gene band of treatment with the corresponding control band relative to positive control* GAPDH* band.

### 2.6. Statistical Analysis

The differences between the treatment groups and the control group were analyzed with a General Liner model using SPSS Statistics 17.0 (Statistical Packages for the Social Sciences, released 23 August 2008). Tukey's multiple comparison test was used to identify which treatments conditions were significantly different from each other at a significance level of *P* < 0.05.

## 3. Results

Data presented in Table 2 shows the effects of dietary AA and FOS on growth performance of broiler chickens. Either feeding AA or FOS tended to increase body weight gain. And feeding the combination of AA and FOS maximally increased the body weight gain among the four experimental diets. On the other hand, feed intake was decreased in all treatment groups, and thus, feed conversion ratio was improved by feeding AA and FOS. In addition, feeding AA and the combination of AA and FOS increased the breast muscle weight of broiler chicks, while feeding FOS not significantly. Abdominal fat weight was decreased by feeding all treatment groups, while liver weight was not influenced by treatment groups except the combination.

Table 3 shows plasma concentrations of total cholesterol (TC), triglyceride (TG), HDL-cholesterol, and LDL cholesterol content. Plasma TC, TC, and LDL were decreased by feeding AA, FOS, and the combination of AA and FOS, while plasma HDL was increased.

Dry matter digestibility (DMD), crude protein utilization (CPU), and crude fiber utilization (CFU) were all improved by feeding AA and FOS, while extract utilization (EEU) was only improved by feeding combination of AA and FOS (Table 4).

**Figure 1:**
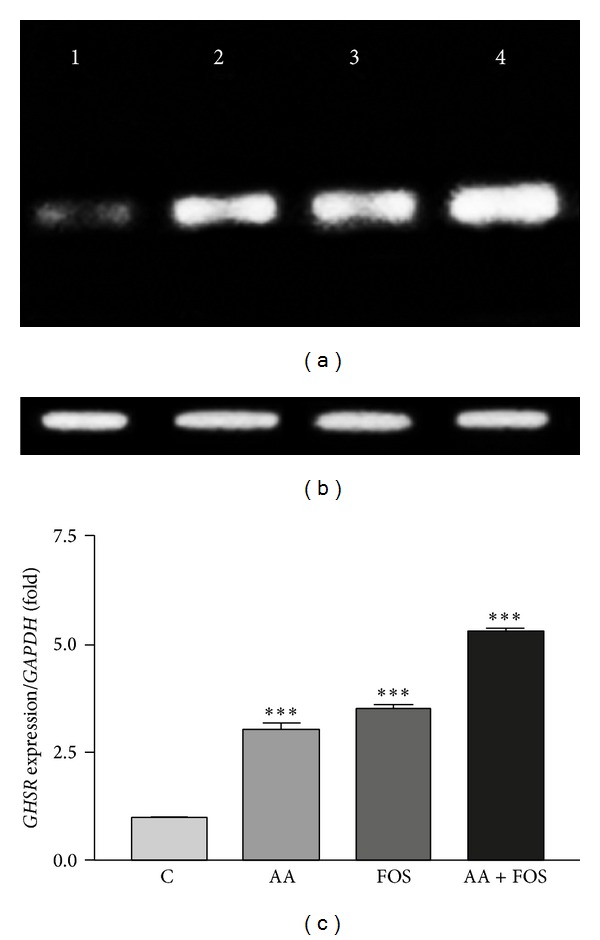
*GHSR* gene expression levels in the skeletal muscles of control and treatment groups. (a) Ethidium bromide stained agarose gel of RT-PCR products* GHSR* gene with size of 533 bp (lane 1: control group and lane 2–4 treatment groups: lane 2, AA group; lane 3, FOS group; lane 4, AA + FOS group) compared to (b) the house-keeping gene,* GAPDH*, with size of 170 bp. (c) Band intensity was quantified using Image J software and the ratio of* GHSR* to* GAPDH* was calculated. Mean ratios of six samples of three experiments performed on different samples and data are expressed as the mean ± SEM and are represented on this figure, relative to the mean ratio of the control group.* GHSR* gene expression levels were significantly higher in treatment groups (AA, FOS, and AA + FOS). ***Significant difference from control group (*P* < 0.001). C = control group, AA =* Aspergillus awamori* group, FOS =* fructooligosaccharide* group, and AA + FOS = combined* Aspergillus awamori* and* fructooligosaccharide* group.

**Figure 2:**
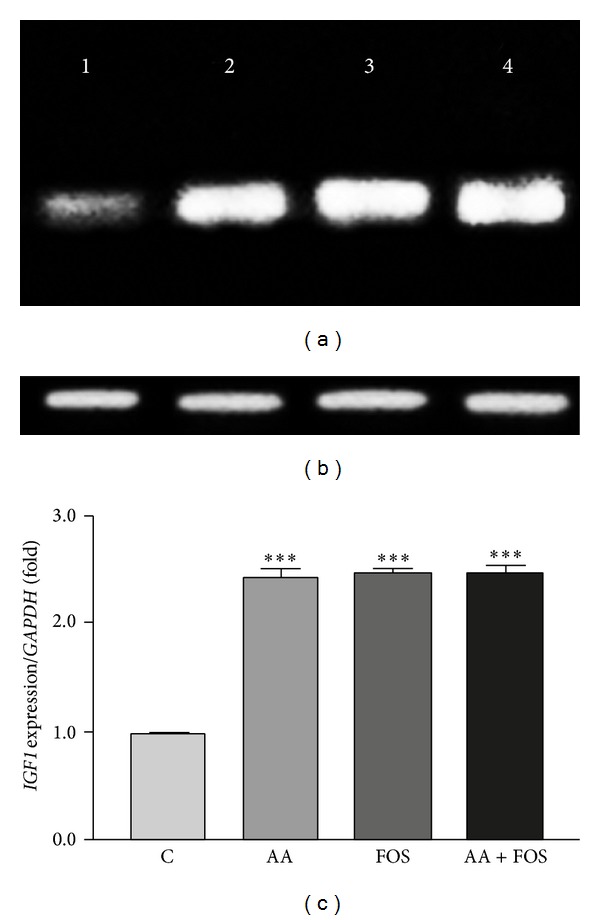
*IGF1* gene expression levels in the skeletal muscles of control and treatment groups. (a) Ethidium bromide stained agarose gel of RT-PCR products* IGF1* gene with size of 583 bp (lane 1: control group and lane 2–4 treatment groups: lane 2, AA group; lane 3, FOS group; lane 4, AA + FOS group) compared to (b) the house-keeping gene,* GAPDH*, with size of 170 bp. (c) Band intensity was quantified using Image J software and the ratio of* IGF1* to* GAPDH* was calculated. Mean ratios of six samples of three experiments performed on different samples and data are expressed as the mean ± SEM and are represented on this figure, relative to the mean ratio of the control group.* IGF1* gene expression levels were significantly higher in treatment groups (AA, FOS, and AA + FOS). ***Significant difference from control group (*P* < 0.001). C = control group, AA =* Aspergillus awamori* group, FOS =* fructooligosaccharide* group, and AA + FOS = combined* Aspergillus awamori* and* fructooligosaccharide* group.

**Figure 3:**
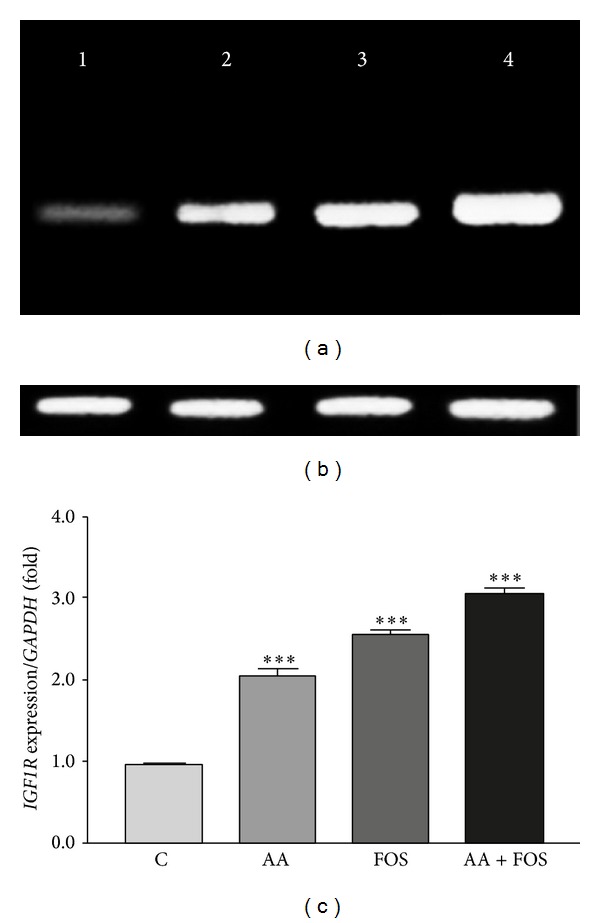
*IGF1R* gene expression levels in the skeletal muscles of control and treatment groups. (a) Ethidium bromide stained agarose gel of RT-PCR products* IGF1R* gene with size of 167 bp (lane 1: control group and lane 2–4 treatment groups: lane 2, AA group; lane 3, FOS group; lane 4, AA + FOS group) compared to (b) the house-keeping gene,* GAPDH*, with size of 170 bp. (c) Band intensity was quantified using Image J software and the ratio of* IGF1R* to* GAPDH* was calculated. Mean ratios of six samples of three experiments performed on different samples and data are expressed as the mean ± SEM and are represented on this figure, relative to the mean ratio of the control group.* IGF1R* gene expression levels were significantly higher in treatment groups (AA, FOS, and AA + FOS). ***Significant difference from control group (*P* < 0.001). C = control group, AA =* Aspergillus awamori* group, FOS =* fructooligosaccharide* group, and AA + FOS = combined* Aspergillus awamori* and* fructooligosaccharide* group.

Changes in transcription levels of* GHSR*,* IGF1*, and* IGF1R* genes in muscles after treatment in comparison to control group and to the housekeeping gene,* GAPDH*, are presented in Figures 1, 2, and 3. Expression of the three genes was remarkably upregulated in treatment groups as compared to control group (Figures 1–3). The mRNAs of* GHSR* and* IGF1R* were increased by feeding AA and FOS and highly significant by the combination of AA and FOS. However, the increased level of* IGF1* mRNA was nearly similar after feeding the three experimental diets.

## 4. Discussion

The main aim of the present study was to show how the growth performance, digestibility, and protein metabolism can be improved by feeding* Aspergillus awamori* (AA) and* fructooligosaccharide* (FOS) and its mechanism in broiler chickens. The combined feeding AA and FOS synergistically promoted the broiler performance. The improvement in weight gain and feed efficiency due to the combination of AA and FOS may be partially due to the increase in metabolic energy of the feed [3] or due to improvement of the survival and implantation of live microbial dietary supplements in the gastrointestinal tract. Those effects are due to activating the metabolism of one or a limited number of health-promoting bacteria or by selectively stimulating their growth, which improved the welfare of the broilers [8]. The* fructooligosaccharide* does not selectively enrich for beneficial bacterial populations. Instead, it is thought to act by binding and removing pathogens from the intestinal tract and stimulation of the immune system [23].

In this study we have found that body weight and breast muscle weight were increased by AA and FOS. Similarly, Yamamoto et al. have noticed a significant increase in carcass weight and breast muscle weight of broilers after feeding on diets containing 0.05 and 1% of AA [24]. This seems to be due to a growth promoting factor produced by* Aspergillus* [25]. Guobin et al. noted that the IGF1 is one of the main growth factors that stimulate protein synthesis in muscle tissue [26]. Also, IGFs are important positive modulators of body and muscle growth in mammals and chickens. However, Beccavin et al. reported that IGFI levels were higher in the fast growing genotype than in the slow growing genotype [27]. The* IGF1* gene exerts anabolic actions on skeletal muscle tissue. These actions include stimulation of amino acids uptake and incorporation into protein, uridine and thymidine synthesis into nucleic acid glucose uptake, cell proliferation, and suppression of protein degradations [28]. All IGF1 actions are mediated by its receptor, IGF1R. Therefore, it is likely that feeding on AA and FOS would have a positive effect on expression of IGF1 and its receptor IGF1R. Indeed, the expression of these two genes was remarkably upregulated in treatment groups as compared to control group. This indicates the activation of pathways related to the skeletal muscle protein synthesis. In consistence, Beccavin et al. have found that chickens with high growth rate show higher circulating levels of* IGFI* and higher* IGFI* mRNA levels as compared to chickens with low growth rate [29]. This supports the hypothesis of Duclos et al. that growth is controlled by a complex interaction of genetic, hormonal, and nutritional factors [30]. In addition, these results support our previous notion that feeding on AA stimulates skeletal muscle growth through decreasing breakdown of their protein [3].

Abdominal fat weight was decreased by feeding AA and FOS. This agrees with Navidshad et al. who found that broilers fed on diets containing* Fermacto*, which is a commercial fermentation product at levels of 0.15 and 0.3%, have a significant lower abdominal fat [31]. We suggested that certain microflora present in gastrointestinal tract of a bird impaired the absorption of cholesterol and bile acid. So it is possible that combination of AA and FOS may cause lower absorption and deposition of fat content around the abdomen. Plasma total cholesterol, triglycerides, and LDL-C were decreased, while plasma HDL-C was increased by the combination of AA and FOS. Kim et al. investigatedthat* Aspergillus oryzae* at 0.1% in diet significantly lowered serum cholesterol and triglyceride in broiler chickens [32]. The mechanism underlying the cholesterol lowering effect of* Aspergillus* could be related to an inhibitor of 3-hydroxyl-3-methylglutaryl-coenzyme (HMG-CoA) reductase [33, 34]. It is well known that the HMG-CoA reductase inhibitor, Statin, which was extracted from a fungus, inhibits the rate-limiting step in cholesterol synthesis. Previous studies have noticed a significant decrease in plasma cholesterol in broiler chickens fed on diets containing two levels of probiotic (0.8 and 1 g/kg) [35] and prebiotics and synbiotics [36].

Utilization coefficients of nutrients dry matter (DM), crude protein (CPU), crude fiber (CFU), and ether extracts (EEU) were all improved by feeding AA and FOS. The broilers do not produce some enzymes such as cellulase and xylanase which are required for the digestion of soluble nonstarch polysaccharides. These enzymes can be produced by* Aspergillus awamori* [2, 3, 37] and thus we thought that the improved digestibility might be due to feeding on AA. It was reported that exogenous enzymes had potential to improve broiler performance [38]. Furthermore,* Aspergillus awamori* possesses the ability to digest raw starches [39]. In this study, we also found a significant improvement in digestibility after feeding on FOS. This improved digestibility may be due to increased activities of amylase, protease, trypsin, and lipase in the small intestine [12]. Some investigators reported that addition of FOS to diets caused significantly greater fecal lipid excretion in rats [40]. Addition of FOS enhanced the growth of* Bifidobacterium* and* Lactobacillus*, which had the action of precipitation and assimilation with bile salt [41] thus increasing fecal bile acid excretion [40, 42] and decreasing its intestinal concentration. Intestinal bile acid has a great impact on the lipid emulsification and the activities of lipase. The growth hormone secretagogue receptor (*GHSR*) is a candidate gene for food intake and food conversion rate and so its absence decreases the food conversion rate and subsequently fat content and body weight [19, 21]. In line with these physiological activities, we have observed a remarkable high expression level of* GHSR* after supplementation of AA and FOS. This elevation is therefore likely to be responsible for, or associated with, improved digestibility and food conversion rate. Further investigations are needed to validate this possibility.

## 5. Conclusions

We concluded that growth performance and digestibility can be improved by supplementation of both* Aspergillus awamori* and* fructooligosaccharide* to the broiler diets probably by promoting skeletal muscle protein metabolism.

## Figures and Tables

**Table 1:** Composition and nutrient analysis of the basal diet.

Ingredients	g/kg
Corn	565.2
Soybean meal, 48%	300.0
Corn gluten meal, 60%	60.0
Premix*	3.0
Soy oil	40.0
Dicalcium phosphate	18.0
Limestone	10.0
Salt	3.8

Calculated values**	
CP, %	21.65
ME, M·J/Kg	13.17
Crude fibre, %	3.05
Ether extract, %	6.6
Ca, %	0.89
P, %	0.48

*Included 3.0 g/kg of vitamin and mineral mix supplied the following per kg of diet: retinyl acetate: 11 000 IU; cholecalciferol: 1810 IU; DL-*α*-tocopheryl acetate: 10.8 mg; menadione sodium bisulphate: 2 mg; riboflavin: 5.7 mg; pyridoxine hydrochloride: 2 mg; cyanocobalamin: 0.025 mg; nicotinic acid: 27 mg; folic acid: 0.48 mg; pantothenic acid: 13 mg; choline chloride: 252 mg; Mn: 100 mg; Zn: 64 mg; Cu: 5 mg; Se: 0.23 mg; I: 0.5 mg and Co:  0.5 mg.

**According to NRC (2003).

**Table 2:** Effect of using *Aspergillus awamori* and *fructooligosaccharide* on growth performances and organ weights in broilers chicks.

	Treatments				ANOVA		
	Control	AA	FOS	AA + FOS	AA	FOS	AA × FOS
Initial body weight (g)	320 ± 4	321 ± 5	320 ± 4	319 ± 6	NS	NS	NS
BWG, (g/22 day)	1569 ± 25^b^	1661 ± 21^ab^	1624 ± 27^ab^	1716 ± 29^a^	NS	NS	∗
FI, (g/22 day)	2924 ± 52^a^	2814 ± 63^b^	2783 ± 68^b^	2734 ± 81^c^	∗	∗	∗∗
FCR	1.86 ± 0.02^a^	1.69 ± 0.02^b^	1.71 ± 0.3^b^	1.59 ± 0.04^c^	∗	∗	∗∗
BMW, (g/100 g BW)	22.9 ± 0.7^b^	27.2 ± 0.7^a^	25.1 ± 1.0^ab^	28.8 ± 1.2^a^	∗	NS	∗∗
Liver, (g/100 g BW)	2.8 ± 0.2^b^	3.3 ± 0.1^a^	3.3 ± 0.1^a^	3.6 ± 0.1^a^	NS	NS	∗∗
Abdominal fat, (g/100 g BW)	1.7 ± 0.05^a^	0.9 ± 0.1^b^	1.0 ± 0.06^b^	0.7 ± 0.05^c^	∗	∗	∗∗

AA and FOS were added to the basal diet at level of 0.05%. Values are expressed as means ± standard error. Data were analyzed by two-way analysis of variance and Duncan's multiple range test. Means within a row not sharing a common superscript significantly differ from each other. NS: not significant (*P* > 0.05); *P < 0.05; **P < 0.01. Body weight gain (BWG), feed intake (FI), feed conversion ratio (FCR), and breast muscle weight (BMW).

**Table 3:** Effect of using *Aspergillus awamori* and *fructooligosaccharide* on blood lipids in broilers chicks.

	Treatments				ANOVA		
	Control	AA	FOS	AA + FOS	AA	FOS	AA × FOS
TC, mg/dL	148 ± 5^a^	125 ± 8^b^	123 ± 8^b^	121 ± 5^b^	∗	∗	NS
TG, mg/dL	28.2 ± 1.4^a^	19.7 ± 1.5^b^	19.3 ± 2.2^b^	19.1 ± 2.0^b^	∗	∗	∗
HDL, mg/dL	77 ± 2^b^	93 ± 2^a^	93 ± 2^a^	95 ± 3^a^	∗	∗	NS
LDL, mg/dL	65 ± 6^a^	25 ± 8^b^	26 ± 8^b^	23 ± 5^c^	∗	∗	∗∗

AA and FOS were added to the basal diet at level of 0.05%. Values are expressed as means ± standard error. Data were analyzed by two-way analysis of variance and Duncan's multiple range test. Means within a row not sharing a common superscript significantly differ from each other. NS: not significant (*P* > 0.05); *P < 0.05; **P < 0.01. Total cholesterol (TC), triglyceride (TG), high density lipoprotein (HDL), and low density lipoprotein (LDL).

**Table 4:** Effect of using *Aspergillus awamori* and *fructooligosaccharide* on utilization coefficients of nutrients in broilers chicks.

	Treatments				ANOVA		
	Control	AA	FOS	AA + FOS	AA	FOS	AA × FOS
DMD, (%)	67.4 ± 6.1^c^	75.5 ± 4.1^a^	72.7 ± 4.6^b^	77.2 ± 4.8^a^	NS	∗	∗
CPU, (%)	66.8 ± 3.1^c^	75.4 ± 5.2^a^	71.5 ± 4.3^b^	75.5 ± 6.3^a^	∗	∗	∗
CFU, (%)	62.5 ± 4.2^b^	66.3 ± 5.2^a^	63.9 ± 4.3^ab^	67.9 ± 4.3^a^	∗	NS	∗
EEU, (%)	56.7 ± 5.3^b^	58.8 ± 4.1^ab^	59.5 ± 6.2^ab^	65.4 ± 3.2^a^	NS	NS	∗

AA and FOS were added to the basal diet at level of 0.05%. Values are expressed as means ± standard error. Data were analyzed by two-way analysis of variance and Duncan's multiple range test. Means within a row not sharing a common superscript significantly differ from each other. NS: not significant (*P* > 0.05); *P < 0.05. Dry matter digestibility (DMD), crude protein utilization (CPU), crude fiber utilization (CFU), and ether extract utilization (EEU).

## References

[1] Fuller R (1989). Probiotics in man and animals. *The Journal of Applied Bacteriology*.

[2] Saleh AA, Eid YZ, Ebeid TA, Kamizono T, Ohtsuka A, Hayashi K (2011). Effects of feeding *Aspergillus awamori* and *Aspergillus niger* on growth performance and meat quality in broiler chickens. *Journal of Poultry Science*.

[3] Saleh AA, Eid YZ, Ebeid TA, Ohtsuka A, Yamamoto M, Hayashi K (2012). Feeding *Aspergillus awamori* reduces skeletal muscle protein breakdown and stimulates growth in broilers. *Animal Science Journal*.

[4] Saleh AA, Hayashi K, Ohtsuka A (2013). Synergistic effect of feeding *Aspergillus awamori* and *Saccharomyces cerevisiae* on growth performance in broiler chickens; promotion of protein metabolism and modification of fatty acid profile in the muscle. *Journal of Poultry Science*.

[5] Pandey A, Nigam P, Soccol CR, Soccol VT, Singh D, Mohan R (2000). Advances in microbial amylases. *Biotechnology and Applied Biochemistry*.

[6] Bigelis R, Lasure L, Beuchat LR (1987). Fungal enzymes and primary metabolites used in food processing. *Food and Beverage Mycology*.

[7] Zubillaga M, Weill R, Postaire E, Goldman C, Caro R, Boccio J (2001). Effect of probiotics and functional foods and their use in different diseases. *Nutrition Research*.

[8] Gibson GR, Roberfroid MB (1995). Dietary modulation of the human colonic microbiota: introducing the concept of prebiotics. *Journal of Nutrition*.

[9] Zimmermann B, Bauer E, Mosenthin R (2001). Pro- and prebiotics in pig nutrition—potential modulators of gut health?. *Journal of Animal and Feed Sciences*.

[10] Kaplan H, Hutkins RW (2000). Fermentation of fructooligosaccharides by lactic acid bacteria and bifidobacteria. *Applied and Environmental Microbiology*.

[11] Wu D, Wu LY (1999). Effects of fructooligosaccharide on the broiler production. *Acta Agriculture Zhejiangensis*.

[12] Xu ZR, Hu CH, Xia MS, Zhan XA, Wang MQ (2003). Effects of dietary fructooligosaccharide on digestive enzyme activities, intestinal microflora and morphology of male broilers. *Poultry Science*.

[13] Fukata T, Sasai K, Miyamoto T, Baba E (1999). Inhibitory effects of competitive exclusion and fructooligosaccharide, singly and in combination, on *Salmonella* colonization of chicks. *Journal of Food Protection*.

[14] Xu Z-R, Hu C-H, Wang M-Q (2002). Effects of fructooligosaccharide on conversion of L-tryptophan to skatole and indole by mixed populations of pig fecal bacteria. *The Journal of General and Applied Microbiology*.

[15] Schrezenmeir J, de Vrese M (2001). Probiotics, prebiotics, and synbiotics—approaching a definition. *The American Journal of Clinical Nutrition*.

[16] Roberfroid MB (1998). Prebiotics and synbiotics: concepts and nutritional properties. *British Journal of Nutrition*.

[17] Duclos MJ, Chevalier B, Goddard C, Simon J (1993). Regulation of amino acid transport and protein metabolism in myotubes derived from chicken muscle satellite cells by insulin-like growth factor-I. *Journal of Cellular Physiology*.

[18] Jennische E, Hansson H-A (1987). Regenerating skeletal muscle cells express insulin-like growth factor I. *Acta Physiologica Scandinavica*.

[19] Mitchell PJ, Johnson SE, Hannon K (2002). Insulin-like growth factor I stimulates myoblast expansion and myofiber development in the limb. *Developmental Dynamics*.

[20] Sun Y, Wang P, Zheng H, Smith RG (2004). Ghrelin stimulation of growth hormone release and appetite is mediated through the growth hormone secretagogue receptor. *Proceedings of the National Academy of Sciences of the United States of America*.

[21] Zigman JM, Nakano Y, Coppari R (2005). Mice lacking ghrelin receptors resist the development of diet-induced obesity. *Journal of Clinical Investigation*.

[22] Abramoff MD, Magelhaes PJ, Ram SJ (2004). Image processing with Image. *Journal of Biophotonics International*.

[23] Spring P, Wenk C, Dawson KA, Newman KE (2000). The effect of dietary mannanoligosaccharides on cecal parameters and the concentrations of enteric bacteria in the ceca of *Salmonella*- challenged broiler chicks. *Poultry Science*.

[24] Yamamoto M, Saleh F, Tahir M, Ohtsuka A, Hayashi K (2007). The effect of Koji-feed (fermented distillery by-product) on the growth performance and nutrient metabolizability in broiler. *Journal of Poultry Science*.

[25] Kamizono T, Nakashima K, Ohtsuka A, Hayashi K (2010). Effects of feeding hexane-extracts of a shochu distillery by-product on skeletal muscle protein degradation in broiler chicken. *Bioscience, Biotechnology and Biochemistry*.

[26] Guobin C, Xiangping L, Jing L (2011). Temporal and spatial expression of the pax-7 gene during chicken embryo and postnatal development. *Journal of Animal and Veterinary Advances*.

[27] Beccavin C, Chevalier B, Simon J, Duclos MJ (1999). Circulating insulin-like growth factors (IGF-I and -II) and IGF binding proteins in divergently selected fat or lean chickens: effect of prolonged fasting. *Growth Hormone and IGF Research*.

[28] Florini JR, Ewton DZ, Magri KA (1991). Hormones, growth factors, and myogenic differentiation. *Annual Review of Physiology*.

[29] Beccavin C, Chevalier B, Cogburn LA, Simon J, Duclos MJ (2001). Insulin-like growth factors and body growth in chickens divergently selected for high or low growth rate. *Journal of Endocrinology*.

[30] Duclos MJ, Beccavin C, Simon J (1999). Genetic models for the study of insulin-like growth factors (IGF) and muscle development in birds compared to mammals. *Domestic Animal Endocrinology*.

[31] Navidshad B, Adibmoradi M, Pirsaraei ZA (2009). Effects of dietary supplementation of Aspergillus originated prebiotic (Fermacto) on performance and small intestinal morphology of broiler chickens fed diluted diets. *Italian Journal of Animal Science*.

[32] Kim SH, Park SY, Yu DJ (2003). Effects of feeding *Aspergillus oryzae* ferments on performance, intestinal microflora, blood serum components and environmental factors in broiler. *Korean Journal of Poultry Science*.

[33] Hajjaj H, Duboc P, Fay LB, Zbinden I, Macé K, Niederberger P (2005). *Aspergillus oryzae* produces compounds inhibiting cholesterol biosynthesis downstream of dihydrolanosterol. *FEMS Microbiology Letters*.

[34] Saleh AA, Ohtsuka A, Hayashi K (2012). The modification of the muscle fatty acid profile by dietary supplementation with *Aspergillus awamori* in broiler chickens. *British Journal of Nutrition*.

[35] Alkhalf A, Alhaj M, Al-Homidan I (2010). Influence of probiotic supplementation on blood parameters and growth performance in broiler chickens. *Saudi Journal of Biological Sciences*.

[36] Piva A (1998). Non-conventional feed additives. *Journal of Animal Feed Science*.

[37] Yamamoto M, Saleh F, Ohtsuka A, Hayashi K (2005). New fermentation technique to process fish waste. *Animal Science Journal*.

[38] Saleh F, Tahir M, Ohtsuka A, Hayashi K (2005). A mixture of pure cellulase, hemicellulase and pectinase improves broiler performance. *British Poultry Science*.

[39] Amsal A, Takigami M, Ito H (1999). Increased digestibility of raw starches by mutant strains of *Aspergillus awamori*. *Food Science and Technology Research*.

[40] Kim M, Shin HK (1998). The water-soluble extract of chicory influences serum and liver lipid concentrations, cecal short-chain fatty acid concentrations and fecal lipid excretion in rats. *Journal of Nutrition*.

[41] Zhan C (1998). The action of decreasing cholesterol of Bifidobacterium: precipitation and assimilation with biliary salt. *Medicine in Abroad: Antibiotic*.

[42] Delzenne NM, Kok N, Fiordaliso M-F, Deboyser DM, Goethals FM, Roberfroid MB (1993). Dietary fructooligosaccharides modify lipid metabolism in rats. *The American Journal of Clinical Nutrition*.

